# White matter microstructure changes in adults with major depressive disorder: evidence from diffusion magnetic resonance imaging

**DOI:** 10.1192/bjo.2023.30

**Published:** 2023-05-29

**Authors:** Gang Wu, Bohui Mei, Xiaofang Hou, Fukun Wang, Chen Zang, Xiaoya Zhang, Zhenjiang Zhang

**Affiliations:** Laboratory of Magnetic Resonance, Zhumadian Second People's Hospital, China; General Committee Office, Zhumadian Second People's Hospital, China

**Keywords:** Magnetic resonance imaging, diffusion, major depressive disorder, brain, white matter

## Abstract

**Background:**

Major depressive disorder (MDD) is a serious psychiatric disorder marked by low mood and anhedonia. Understanding the neural mechanism of MDD is essential for the treatment of depression. White matter fibres, connecting different computational units in the brain, have an important effect on brain function; however, the mechanism of white matter fibre abnormality in MDD is still unclear.

**Aims:**

Our study expected to find white matter abnormalities associated with the frontal lobe and hippocampus in individuals with MDD.

**Method:**

Using diffusion tensor imaging data and tract-based spatial statistics, we investigated the microstructural differences in white matter fibre tracts between 30 adults with MDD compared with 31 healthy controls, and calculated the association between MDD-related microstructural changes and illness duration.

**Results:**

It was found that patients with MDD showed reduced fractional anisotropy in the genu and body of the corpus callosum, right corona radiata and part of the thalamic radiations, suggesting lower fibrous myelination levels in these regions; the decreased fractional anisotropy in these regions was associated with longer illness duration.

**Conclusions:**

Our results suggest that MDD may be associated with microstructural damage of key fibre tracts, which could provide insights into the understanding and treatment of MDD.

Major depressive disorder (MDD) is a serious mental disorder marked by low mood and anhedonia that lasts for more than 2 weeks. In addition to severe physical and mental pain, MDD can cause decline in cognitive and social function, and is often accompanied with a series of vegetative symptoms such as disturbed appetite and/or sleep. MDD affects about 6% of adults worldwide each year^1^ and is the second largest contributor to the burden of chronic disease, such as diabetes, heart disease and stroke,^2^ and it can also lead to suicide. It is estimated that up to 50% of suicides occur in people with depression, and people with MDD are at a >20 times greater risk of suicide than general population.^3^ Studying the neural mechanism of MDD is of great significance for our understanding and effective treatment of MDD.

Cerebral tissue can be divided into grey matter and white matter, where grey matter is composed of neuronal cell bodies, which are the basic computational units of the central nervous system, whereas white matter is composed of axons connecting different nearby areas, which form the basis of complex cognitive activities by combining different computational units into neural networks.^4^ Most studies on the neural basis of MDD are related to the shape or function of grey matter. For example, previous studies have shown that people with MDD exhibit abnormalities in the dorsolateral prefrontal cortex, related to cognitive control; the subgenual cingulate cortex, related to emotion regulation; and the hippocampus, related to episodic memory formation.^5^ However, in fact, complex cognitive and emotional functions involve multiple brain regions and function in the form of a network, so it is necessary to study the role of white matter connecting different brain regions in MDD. Diffusion tensor imaging (DTI) is the most widely used technique for investigating brain white matter, and uses preferential diffusion measures like fractional anisotropy to quantify microstructural integrity of white matter fibres.^6^ Some DTI studies have investigated the white matter characteristics of MDD.^7,8^ However, the current DTI research has some limitations. First, because DTI image registration often relies on affine transformation of T1-weighted image, which is mainly oriented to grey matter, there is a distortion problem in the white matter region, which may affect the accuracy of the registration. In addition, the disease process of MDD is a dynamic process, and the white matter may not be stable during the development of the disease. However, there are few studies related to the association between the time course of MDD and white matter changes.

## Objective

Tract-based spatial statistics (TBSS) is a DTI-based analysis method that reduces registration distortion by registering white matter to the white matter skeleton,^9^ which is beneficial for obtaining more stable white matter markers of MDD.^10^ Koshiyama et al used TBSS to look at white matter in 398 individuals with MDD and investigated correlations with illness duration.^11^ A meta-analysis Wise et al reported a significant decrease in fractional anisotropy in the genu of the corpus callosum in depression.^12^ In this study, we attempted to characterise the white matter microstructure abnormalities of MDD based on DTI data and using the TBSS method, and found a potential association between these abnormalities and the disease progression of MDD in a Chinese population, so as to provide insights on white matter connectivity in the study and treatment of MDD. Given the results of previous grey and white matter studies, we expected to find white matter abnormalities associated with the frontal lobe and hippocampus.

## Method

### Ethical approval

The authors assert that all procedures contributing to this work comply with the ethical standards of the relevant national and institutional committees on human experimentation and with the Helsinki Declaration of 1975, as revised in 2008. All procedures involving human patients were approved by the Medical Ethics Committee of Zhumadian Second People's Hospital in Henan Province (approval number IRB-2020-006-02). All participants provided written informed consent before participation.

### Participants

Thirty-two patients diagnosed with MDD at Zhumadian Second People's Hospital in Henan Province were recruited. The exclusion criteria were as follows: any history of neurological diseases, intellectual disability, other physical diseases or comorbidities of other disorders; any other mental disorders; pregnancy or breastfeeding; and head trauma resulting in loss of consciousness. For the control group, 32 age- and gender-matched healthy participants were recruited. The healthy controls had no history of mental illness or severe physical illness, and no family history of mental illness.

MDD was diagnosed according to the DSM-5 criteria. All patients were recruited during a depressive episode, which was diagnosed by two professional and experienced psychiatrists. The patients taking medication were on a stable dose for at least 6 weeks or were unmedicated for at least 4 weeks.

### Data acquisition and processing

All magnetic resonance imaging data were obtained with a 3 T Trio scanner with a standard head coil at the Medical Ethics Committee of Zhumadian Second People's Hospital in Henan Province. A spin-echo planar imaging sequence was used to collect the DTI data with the following scan parameters: 32 diffusion directions, *b* = 1,000 s/mm^2^, repetition time 13 000 ms, time to echo 86.1 ms, flip angle 180°, 47 contiguous axial slices, 3 mm thickness, without gap, imaging matrix 128 × 128, field of view 256 × 256 mm^2^.

### DTI analysis

To investigate the alterations of structural diffusion features of white matter in patients with MDD, the DTI data were analysed with the FMRIB Software Library (FSL) for Windows (Analysis Group, Oxford, UK; see www.fmrib.ox.ac.uk/fsl). First, nonbrain tissues were removed from the DTI data by using the brain extraction tool algorithm in FSL. Next, head motion and eddy current corrections were carried out by the affine transformation between the gradient images and the baseline *b* = 0 image. Then, diffusion tensors were calculated with the *dtifit* tool in FSL, and fractional anisotropy, mean diffusivity, axial diffusivity and radial diffusivity maps were obtained. Since fractional anisotropy is the most widely used in DTI studies and is used as a customary indicator in TBSS studies,^13,14^ we chose fractional anisotropy in this study for follow-up research. All patients’ fractional anisotropy maps were aligned with the Montreal Neuroimaging Institute (MNI152) template space,^15^ using the non-linear registration tool FNIRT.

A voxel-wise statistical analysis of the fractional anisotropy map between MDD and healthy control groups was performed with TBSS pipelines^9^ (https://fsl.fmrib.ox.ac.uk/fsl/fslwiki/TBSS). MNI-space fractional anisotropy maps of all patients were used to generate a mean fractional anisotropy map. Then, the fractional anisotropy maps of all patients were projected onto a skeletonised fractional anisotropy map derived from the mean fractional anisotropy image, which was thresholded by 0.2.

### Statistical analysis

For continuous variables, two-sample *t*-tests were used to test group difference between MDD and control groups. The *χ*^2^-test was used to test the group difference in gender proportion. Two-sample *t*-tests were used to test for fractional anisotropy differences in voxel levels between the MDD and healthy control groups. The voxel-wise comparisons of within-skeleton DTI features were performed via permutation-based non-parametric testing with 5000 permutations, with age and gender included as nuisance covariates. The statistical significance was set at *P* < 0.05 after adjusting for multiple comparisons, using the threshold-free cluster enhancement (TFCE) method in FSL Randomize.^16^ In a previous paper comparing the effect of different multiple comparison correction strategies on the reproducibility of results, TFCE combined with permutation-based testing has been proven to be the most reproducible cross-sample among various strategies, which is very suitable for improving the credibility of neuroimaging studies with small samples.^17^ To avoid noise, only clusters with voxel number >300 are reserved. The values and coordinates of the peak points of each cluster were reported.

To test whether MDD-related white matter abnormalities were significantly associated with illness duration in MDD, we used linear regression models to examine the association between mean fractional anisotropy values of white matter clusters exhibiting significant abnormalities in MDD and self-reported illness duration, measured by month. A total of 19 participants (seven males, age 35.2 ± 7.86 years, illness duration 37.72 ± 47.69 weeks) of 30 patients with MDD reported exact illness duration. Linear regression analysis was performed based on the *lm* function in the R version 4.1.0 (R Foundation for Statistical Computing) built-in package *psych* (https://statisticsglobe.com/psych-r-package), in which the fractional anisotropy value of each cluster was used as independent variable; age, gender and body mass index were used as covariates; and illness duration was used as dependent variable. The model provides a *β*-value and its corresponding *t*- and *P*-values for each independent variable, which are generated based on one-sample *t*-tests to detect whether *β* is significantly greater than or less than 0. In the regression analyses, multiple comparative corrections were made (family-wise error method, *N* = 3 clusters).

## Results

The mean (±s.d.) age of the MDD group was 35.67 ± 9.47 years, and 56.67% of the patients were female (17 males, 30 females). The mean (±s.d.) age of the healthy control group was 36.53 ± 9.21 years, 58.1% of the healthy controls were female (18 males, 30 females). No significant differences in age and gender were observed between the two groups (*P* > 0.05). In the MDD group, the mean (±s.d.) duration of illness was 35.55 ± 47.81 weeks, and the mean (±s.d.) body mass index was 22.63 ± 2.82 kg/m^2^. The participant characteristics are presented in Table 1.

**Table 1:** Basic information of the included participants

Variable	Major depressive disorder (*n* = 30)	Healthy controls (*n* = 31)
Age, years, mean ± s.d.	35.67 ± 9.47	36.53 ± 9.21
Gender, female/male	17/13	18/13
Duration of illness in months, mean ± s.d.	35.55 ± 47.81	——
Body mass index, kg/m^2^, mean ± s.d.	22.63 ± 2.82	——

**Fig. 1:**
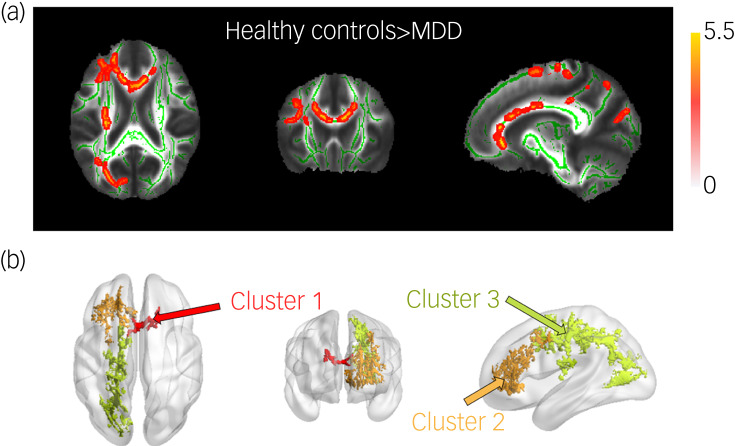
(a) *t*-value map of fractional anisotropy intergroup difference between healthy control and MDD groups. Only voxels that passed the permutation test in combination with threshold-free cluster enhancement were retained (*P* < 0.05). (b) Three clusters with significant fractional anisotropy differences between healthy control and MDD groups. Cluster 1 mainly covered the body of the corpus callosum and a part of genu of the corpus callosum, cluster 2 covered the genu of the corpus callosum and the right anterior corona radiata, and cluster 3 mainly covered the right superior and posterior corona radiata and right posterior thalamic radiation. MDD, major depressive disorder.

As shown in Table 2 and Figure 1, We found three clusters that passed the TFCE-based permutation test, in which cluster 1 mainly covered the body of the corpus callosum and a part of the genu of the corpus callosum (peak *t* = 3.71, *P* = 0.0442), cluster 2 covered the genu of the corpus callosum and part of the right anterior corona radiata (*t* = 3.88, *P* = 0.0318), and cluster 3 mainly covered the right superior and posterior corona radiata and right posterior thalamic radiation (*t* = 5.07, *P* = 0.0102).

**Table 2:** Brain regions where fractional anisotropy was different between individuals with major depressive disorder and healthy controls

Fibres	ID	Peak MNI coordinates					Cluster size (mm^3^)
		*t*-value	*P*-value	*x*	*y*	*z*	
BodyCC	Cluster 1	3.71	0.0442	10	21	19	380
GenuCC + ACR	Cluster 2	3.88	0.0318	−6	25	−1	2914
SCR + PCR + PTR + BodyCC	Cluster 3	5.07	0.0102	−18	−22	57	4558

BodyCC, body of the corpus callosum; GenuCC, genu of the corpus callosum; ACR, Anterior corona radiata; SCR, superior corona radiata; PCR, posterior corona radiata; PTR, posterior thalamic radiation (including optic radiation).

**Fig. 2:**
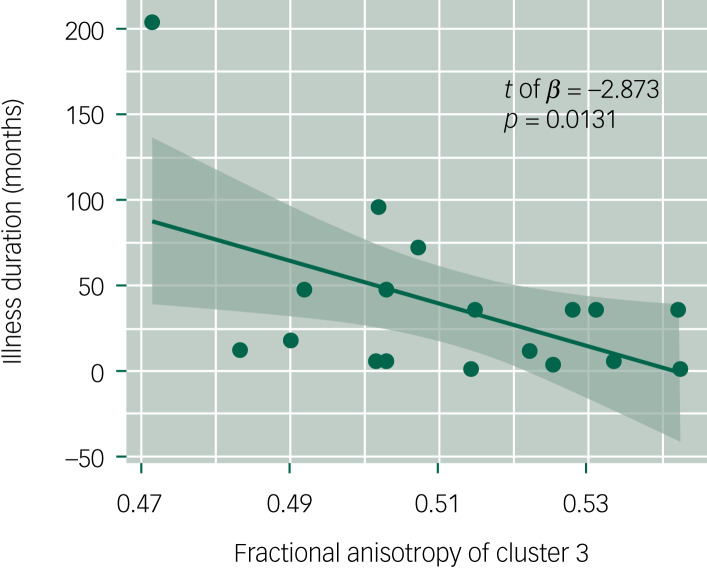
Regression analysis of cluster 3 and illness duration. Each scatter represents a participant, the horizontal axis represents the fractional anisotropy value of cluster 3, the vertical axis represents the illness duration measured in months and the fitted line is based on linear regression. This result was corrected by multiple comparisons (family-wise error method, *P* = 0.0131, which is less than 0.05/3).

In the white matter fractional anisotropy and illness duration association analysis, we found that the fractional anisotropy of cluster 3 showed a significant negative correlation with the disease course (*β* = −1455.33, *t* = −2.873, *P* = 0.0131, which is less than 0.05/3); that is, the lower the fractional anisotropy of cluster 3, the longer the duration of MDD. Fractional anisotropy in the other two clusters did not show a significant correlation with disease duration (Table 3 and Fig. 2).

**Table 3:** Association between fractional anisotropy of different clusters and illness duration in individuals with major depressive disorder

	*β*-value	s.e.	*t*-value	*P*-value
Cluster 1	−469.55	285.47	−1.645	0.124
Cluster 2	−947.1	498.33	−1.901	0.0798
Cluster 3	−1455.33	506.56	−2.873	0.0131

## Discussion

In this study, we used TBSS-styled DTI analysis technology to investigate the microstructural changes in the white matter skeleton of adult patients with MDD compared with healthy controls, and the association between these changes and illness duration. We found that patients with MDD showed reduced fractional anisotropy, which is consistent with the meta-analysis results by Wise et al.^12^ The findings suggested impaired microstructural integrity in the genu and body of the corpus callosum, coronal radiation and thalamic radiation compared with healthy individuals. Further, lower fractional anisotropy in the right superior/anterior corona radiata and posterior thalamic radiation was associated with longer illness duration.

Our results show that the corpus callosum microstructural integrity is impaired in patients with MDD, especially in the anterior (genu) and middle (body) of the corpus callosum. In line with our results, an ENIGMA Major Depressive Disorder Consortium study found that MDD exhibited the largest differences in the corpus callosum and corona radiata.^7^ The genu and body of the corpus callosum mainly connect bilateral rostral cerebral regions (especially the prefrontal cortex and parietal lobe), and is thought to be involved in interhemispheric inhibition of these brain regions.^18,19^ The prefrontal cortex is related to high-order functions such as cognition^20^ and emotion,^21,22^ and makes up the frontoparietal network together with the parietal lobe, which plays an important role in executive control and emotion regulation,^23,24^ both of which are dysfunctional in MDD.^5^ According to previous electroencephalograph studies, the right frontal cortex is associated with negative affects/avoidance motivation, whereas the left frontal cortex is associated with positive affects/approach motivation.^25,26^ Therefore, the reduced microstructural integrity of the genu and body of the corpus callosum may suggest an inability of the left frontal lobe to effectively inhibit the right frontal lobe, leading to emotional imbalance and the dominance of negative emotions.

Several previous studies have reported an association between increased disease duration and reduced fractional anisotropy, particularly in the corpus callosum.^27,28^ However, in our study, in addition to the corpus callosum, white matter fractional anisotropy in the anterior and superior corona radiata and posterior thalamic radiation was found to be reduced in MDD. Both the corona radiata and thalamic radiation are part of the limbic-thalamo-cortical circuitry, which plays an important role in emotion regulation.^29–31^ The anterior and superior corona radiata connect the internal capsule and cortex,^32^ and decreased fractional anisotropy in these tracts might mean that the control function of the frontoparietal cortex to subcortical areas is affected. A large, cross-site DTI study has reported reduced fractional anisotropy in the corona radiata in patients with MDD. Reduced fractional anisotropy in the corona radiata has also been observed in adults with MDD,^33^ elderly patients with MDD^34,35^ and veterans with post-traumatic stress disorder.^31^ A previous study on bipolar depressive disorder also found that the effect of illness duration on processing speed, verbal memory and visual memory was mediated by the fractional anisotropy values of the bilateral corona radiata and genu of the corpus callosum.^36^ The posterior thalamic radiation connects the caudal parts of the thalamus with the occipital/parietal lobe,^37^ and reduced fractional anisotropy in the posterior thalamic radiation has also been reported in previous MDD studies.^38^ Given the role of the thalamus in the motivation circuit and emotion processing, dysfunction of the thalamic radiation might be associated with motivational deficits^39^ or facial recognition deficits in MDD.^40^

In addition to the differences between MDD and healthy control groups, this study found that the fractional anisotropy of the anterior and superior corona radiata decreased with the increasing course of disease in patients with MDD. The negative correlation between illness duration and fractional anisotropy has been reported in previous studies,^27^ and is thought to reflect the destruction of oligodendrocytes by inflammatory factors related to chronic psychological stress.^41,42^ The reduced fractional anisotropy may also be a result of the disruption of circadian rhythms associated with depression; for example, insomnia is a common symptom of depression, and previous studies have found that sleep deprivation causes decreased white matter fractional anisotropy.^43^ In addition, the decreased fractional anisotropy may also be a result of the effects of antidepressant drugs. These questions need to be further explored in future studies.

The cumulative effect of increased illness duration may suggest a continued deterioration of white matter condition in MDD. Although ageing is accompanied by a decrease in fractional anisotropy levels,^44^ given that we controlled for age as a covariate in our analyses, we think that the decline in fractional anisotropy is an effect of illness duration itself. However, the current study cannot rule out the possibility that antidepressants influence the results,^45^ which still needs to be explored further. Moreover, several limitations in our study must be addressed. Body mass index information was only collected in the patient group, whereas patients’ Hamilton Rating Scale for Depression scores and current medication information, and the education level for all participants, were not available from our collected data. These variables should be considered as covariates for the linear regression in future studies. Another limitation is that the sample size is relatively small in the psychiatric neuroimaging studies.

In conclusion, we used DTI analysis technology to investigate the microstructural changes in the white matter skeleton of adult patients with MDD compared with healthy participants, and the association between these changes and illness duration. We found that patients with MDD showed reduced fractional anisotropy, suggesting impaired microstructural integrity and lower myelinisation, in the genu and body of the corpus callosum, coronal radiation and thalamic radiation compared with healthy individuals, and lower fractional anisotropy in the right superior/anterior corona radiata and posterior thalamic radiation was associated with longer illness duration. Our results suggest that MDD may be associated with microstructural damage of key fibre tracts, which could provide insights into the understanding and treatment of MDD.

## Data Availability

The data that support the findings of this study are available from the corresponding author, F.W., upon reasonable request.
